# Supplementary material for: Targeting ubiquitin signaling vulnerabilities in KEAP1-inactivated lung cancer
Source: EMBO J. 2026 Mar 20;45(9):3276–305. doi: 10.1038/s44318-026-00737-9 (PMC13144482; doi:10.1038/s44318-026-00737-9)
Supplement: Supplementary file 1 — Appendix [file 44318_2026_737_MOESM1_ESM.pdf]

Appendix for:

Targeting Ubiquitin Signaling Vulnerabilities in KEAP1-Inactivated Lung Cancer

Table of contents:

Appendix Figure S1	Page 2
Appendix Figure S2	Page 3
Appendix Figure S3	Page 4
Appendix Figure S4	Page 5
Appendix Figure S5	Page 7
Appendix Figure S6	Page 9
Appendix Figure S7	Page 10

Appendix Figure S1:

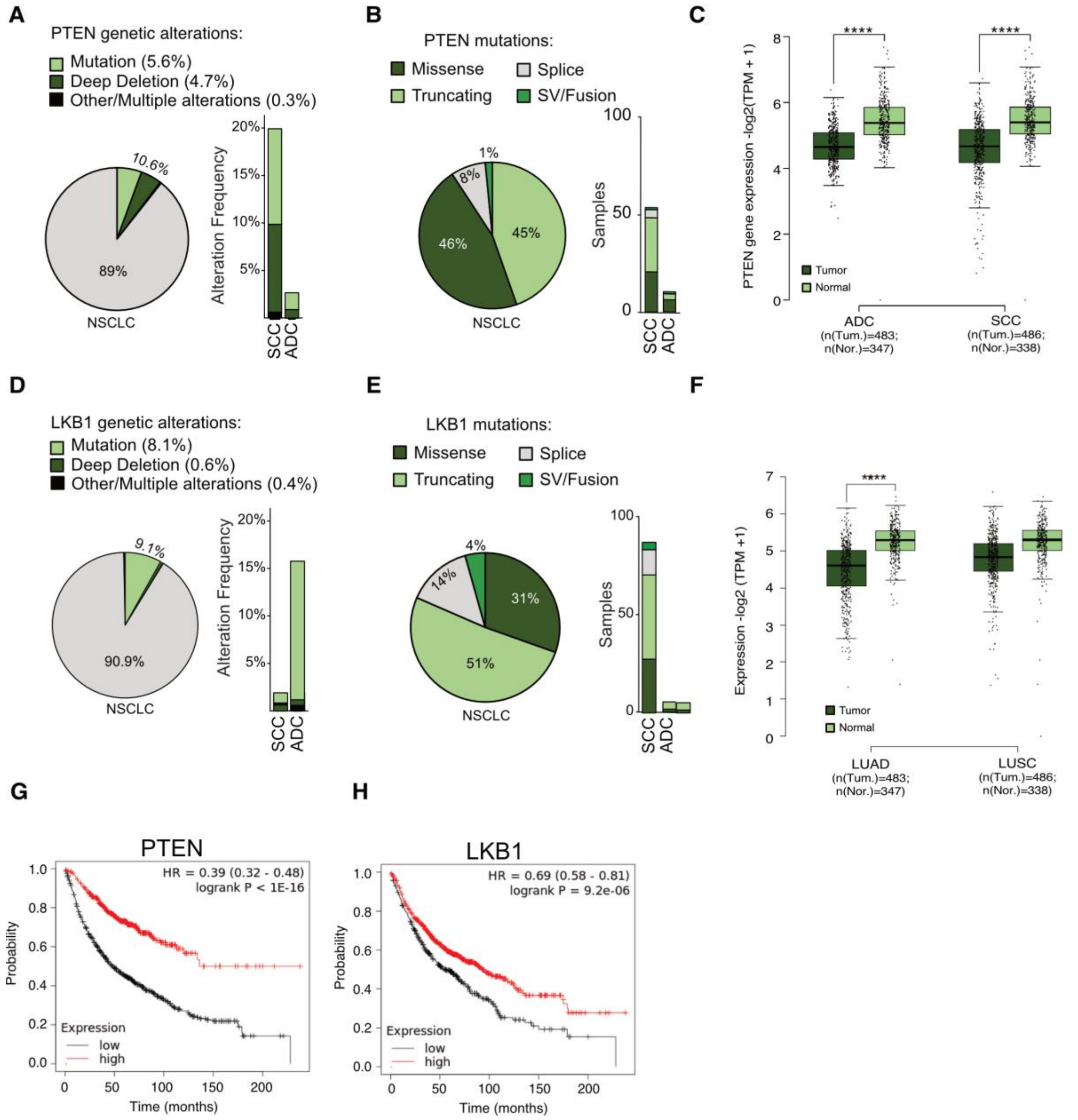

Appendix Figure S1: Genetic alteration landscape of PTEN, LKB1 and UPS components in NSCLC.

A. Genetic alteration of PTEN in a NSCLC (cBioPortal.org).

B. Mutational landscape of PTEN in a SCC and ADC (cBioPortal.org).

C. PTEN mRNA expression in LUAD and LUSC patient samples compared to normal tissue. Data from gepia2.cancer-pku.cn

D. Genetic alteration of LKB1 in a NSCLC (cBioPortal.org).

E. Mutational landscape of LKB1 in a SCC and ADC (cBioPortal.org).

F. LKB1 mRNA expression in LUAD and LUSC patient samples compared to adjacent normal tissue. Data from gepia2.cancer-pku.cn

G, H. Kaplan-Meier curves of overall survival (OS) of NSCLC patients based on PTEN (G) and LKB1 (H) gene expression levels.

Appendix Figure S2:

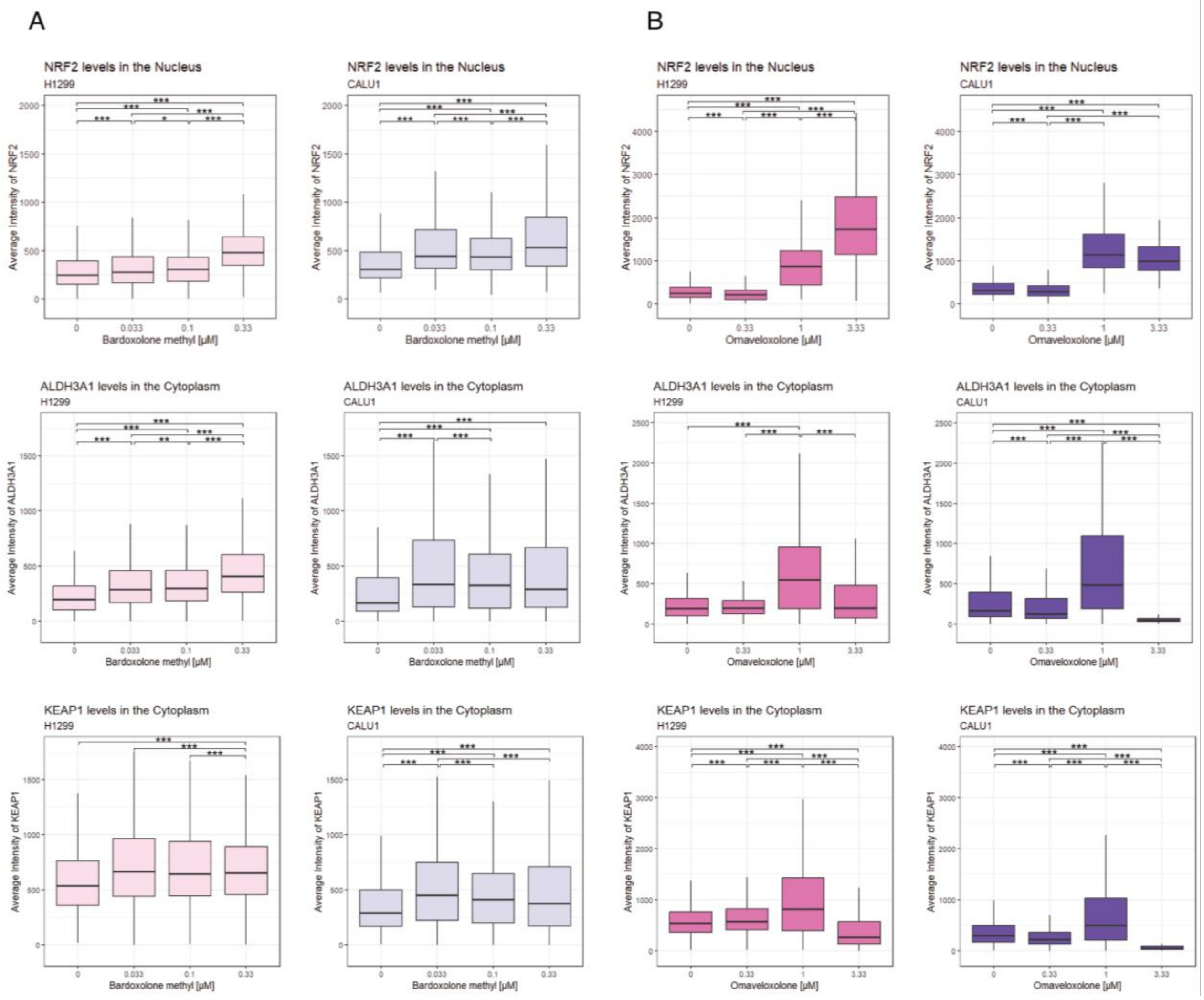

Appendix Figure S2: Omaveloxolone and Bardoxolone Methyl increase NRF2 and Aldh3a1 protein levels.

A and **B**. CALU1 and H1299 were treated with increasing concentrations of Bardoxolone methyl (A) or Omaveloxolone (B) (0 – 0.33 µM) for 24h. Cells were stained for KEAP1, NRF2 and ALDH3A1 and analysed using a high content screening platform. Analysis of the average immunofluorescence intensity (n = 500-5000 cells). ***P ≤ 0.001, **P ≤ 0.01, *P ≤ 0.05. Statistical significance was calculated using Kruskal-Wallis test with a posthoc Dunn's test.

Appendix Figure S3:

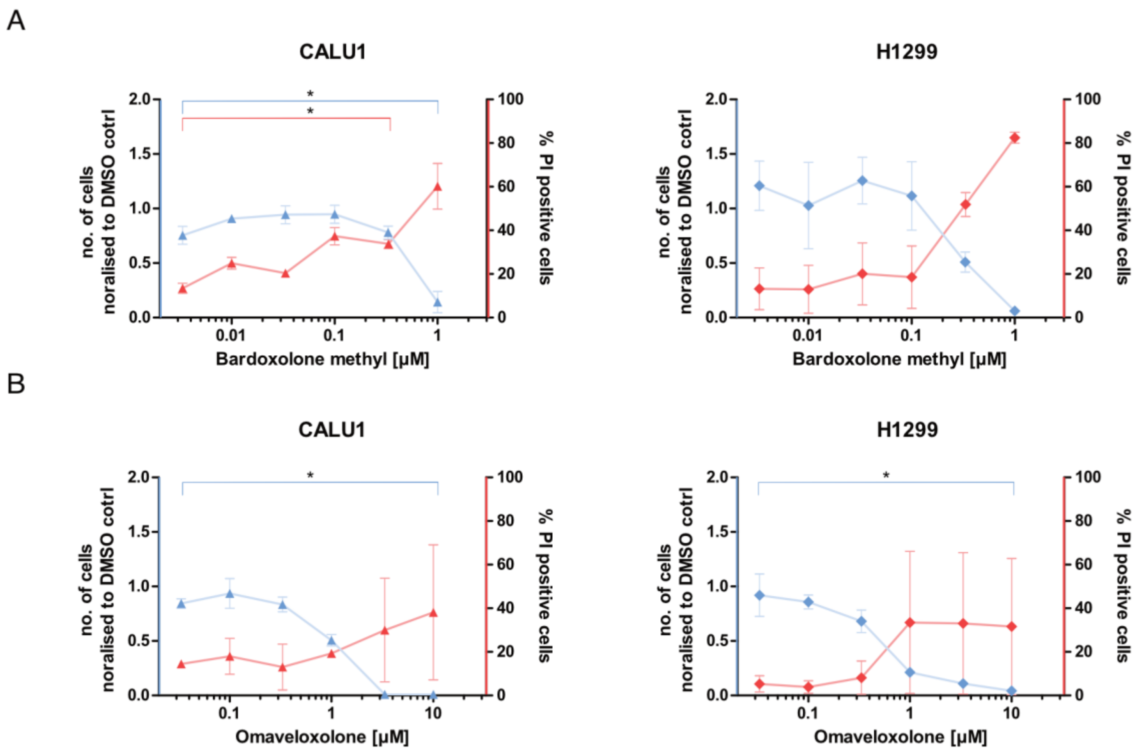

Appendix Figure S3: Impact of KEAP1 inhibitors on NSCLC cell viability.

A and **B**. Live/dead staining of NSCLC cell lines. CALU1 and H1299 were treated with increasing concentrations of Bardoxolone methyl (0 – 1 µM) (A) and Omaveloxolone (0 – 10 µM) (B) for 24h. Cells were stained with Propidium iodide and analysed using a high content screening platform. $*P \leq 0.05$. Statistical significance was calculated using Kruskal-Wallis test with a posthoc Dunn's test.

.

Appendix Figure S4:

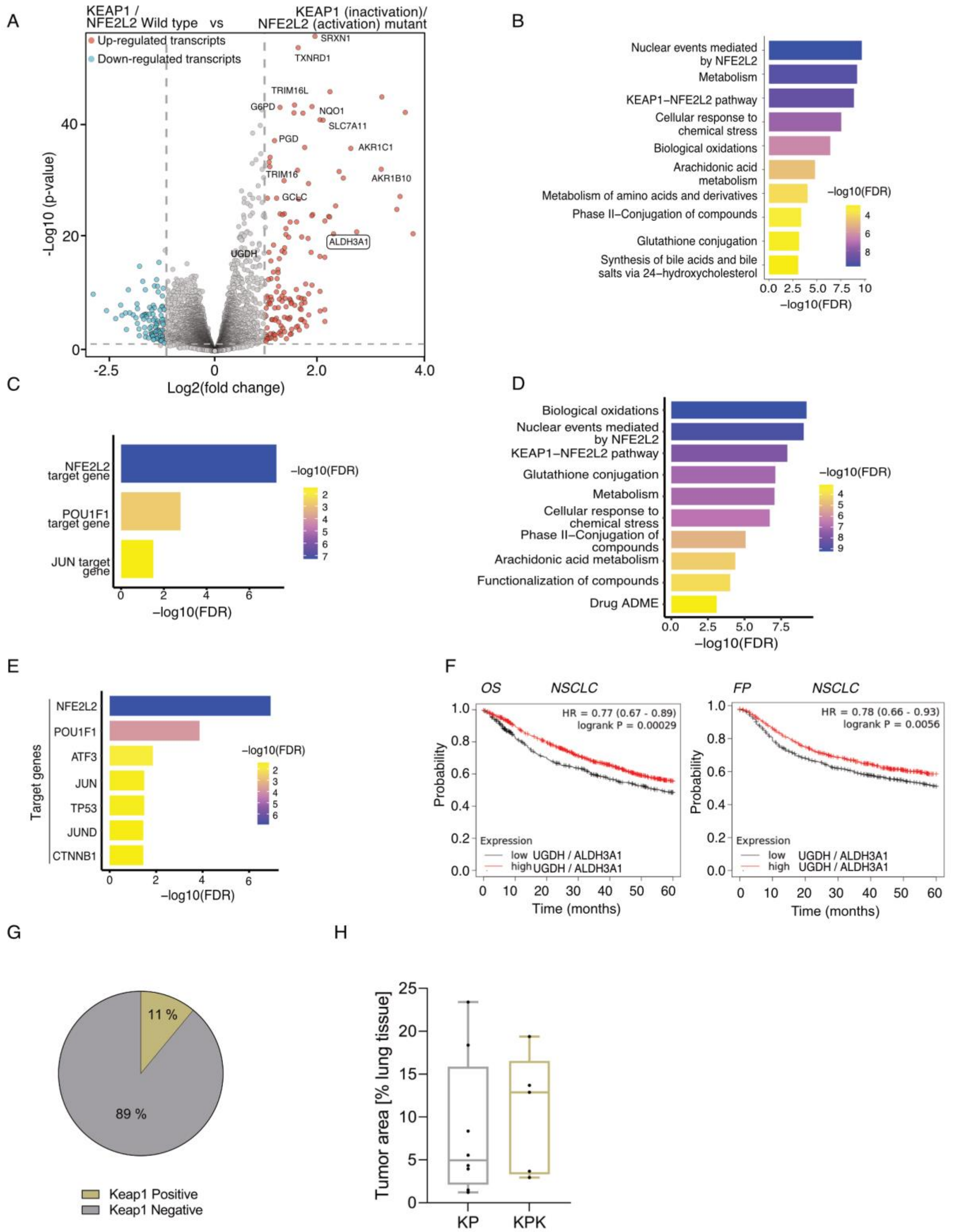

Appendix Figure S4: Oncogenic stress suppress tumor growth *via* reductive stress.

A. Volcano plot displaying transcriptomic signature in wild-type and KEAP1 (inactivation)/NFE2L2 (activation) mutant LUSC patient samples, (n= 349, KEAP1/NFE2L2 WT; n=117, KEAP1/NFE2L2 mutant). Data were obtained from Arolt et al 2023.

B. Reactome pathway analysis from transcriptome reveals activation of NRF2 driven redox reprogramming in KEAP1 (inactivation)/NFE2L2 (activation) mutant LUSC patient samples.
C. Transcription factor analysis from transcriptome reveals NFE2L2 as the top transcription factor affected by KEAP1 (inactivation)/NFE2L2 (activation) mutations in LUAD patient samples (B and C data were obtained from Arolt et al 2023).
D. Reactome pathway analysis from transcriptome reveals activation of NRF2 driven redox reprogramming in KEAP1 (inactivation)/NFE2L2 (activation) mutant LUSC patient samples.
E. Transcription factor analysis from transcriptome reveals NFE2L2 as the top transcription factor affected by KEAP1 (inactivation)/NFE2L2 (activation) mutations in LUSC patient samples. (D and E - data were obtained from Arolt et al 2023).
F. Kaplan-Meier curves of five-year free progression (FP) and overall (OS) survival rates of lung cancer patients based on UGDH and ALDH3A1 gene expression levels. Kmplot.com.
G. Keap1 IHC levels in KPK to demonstrate somatic mutation and loss of Keap1 in KPK tumors.
H. Quantification of tumor area in KP and KPK animals.

Appendix Figure S5:

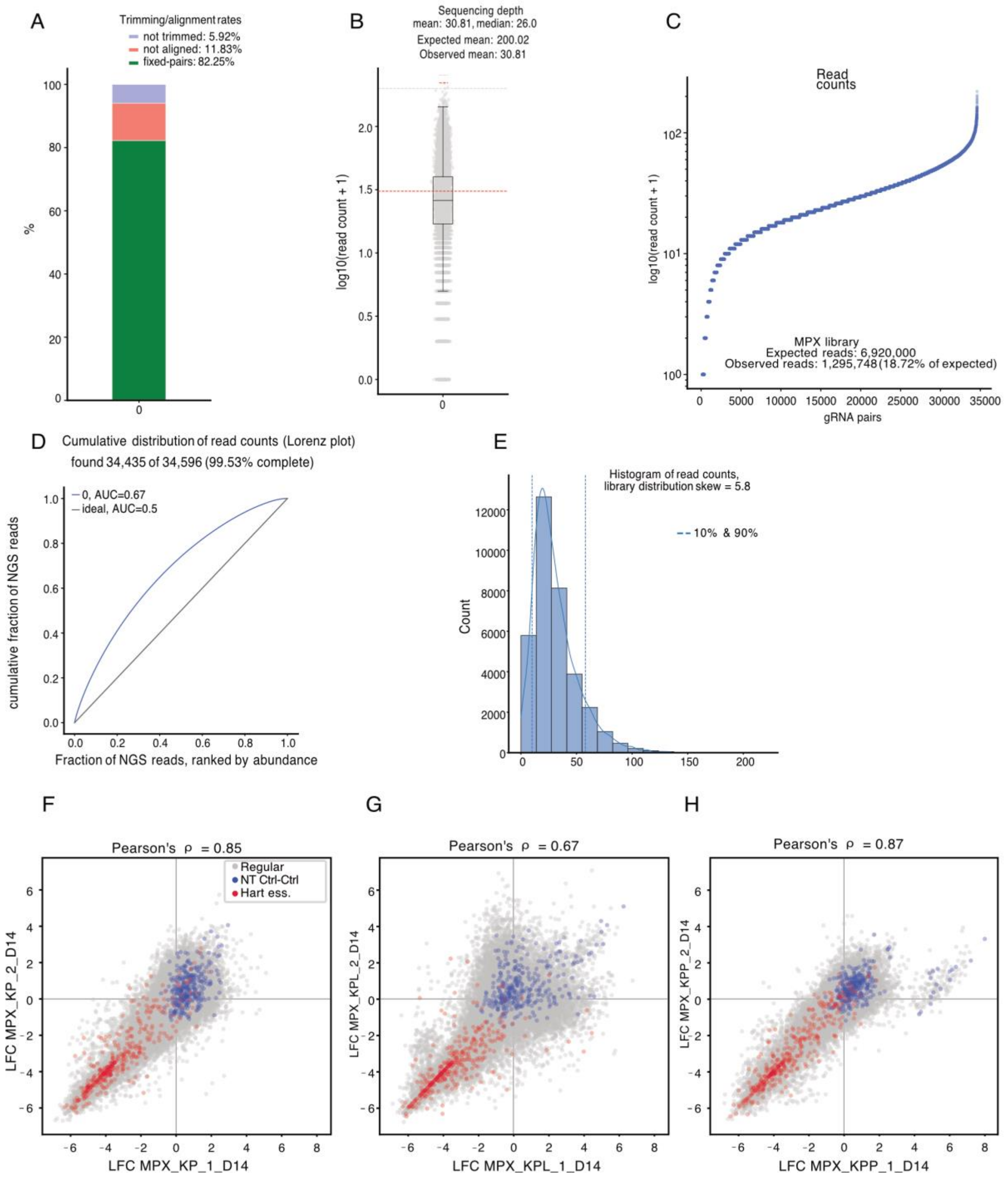

Appendix Figure S5: Generation of UPS centric multiplex CRISPR/Cas9 reagent.

A-E. ReCo plot panel (Wegner et al., 2023). **A** The stacked bar chart indicates the ratio of aligned (green color), not aligned (red color), and not trimmed (light blue color) reads. **B** Box plot displaying the distribution of read counts on a logarithmic scale, the expected and observed mean read counts are highlighted with horizontal dashed lines in gray and red color, respectively. **C** Log-transformed distribution of read counts per gRNA, sorted increasingly. **D** Lorenz curve displaying the cumulative fraction of represented NGS reads versus the gRNAs ranked by abundance of each library revealed a uniform distribution of gRNA sequences. Area under the curve values (AUC) confirm the uniform gRNA

distribution of these libraries. A uniformly distributed library (ideal) is shown in grey. **E** Histogram of gRNA abundance and overlay of the corresponding density plot shows the skew of the NGS read count distribution. The 10 and 90 percentiles are indicated with vertical dashed lines, and the distribution skew of the sample is 5.8.

F-H. The E3-DUB gRNA multiplex screens are highly reproducible (Pearson's correlation coefficient >0.6), as visualized by scatter plots comparing biological replicates (Exp#1/Exp#2). Shown are normalized gRNA read counts at the 14-day time point in multiplex CRISPR/Cas9 screens for KP (F), KPL (G) and KPP (H) cell lines.

Appendix Figure S6:

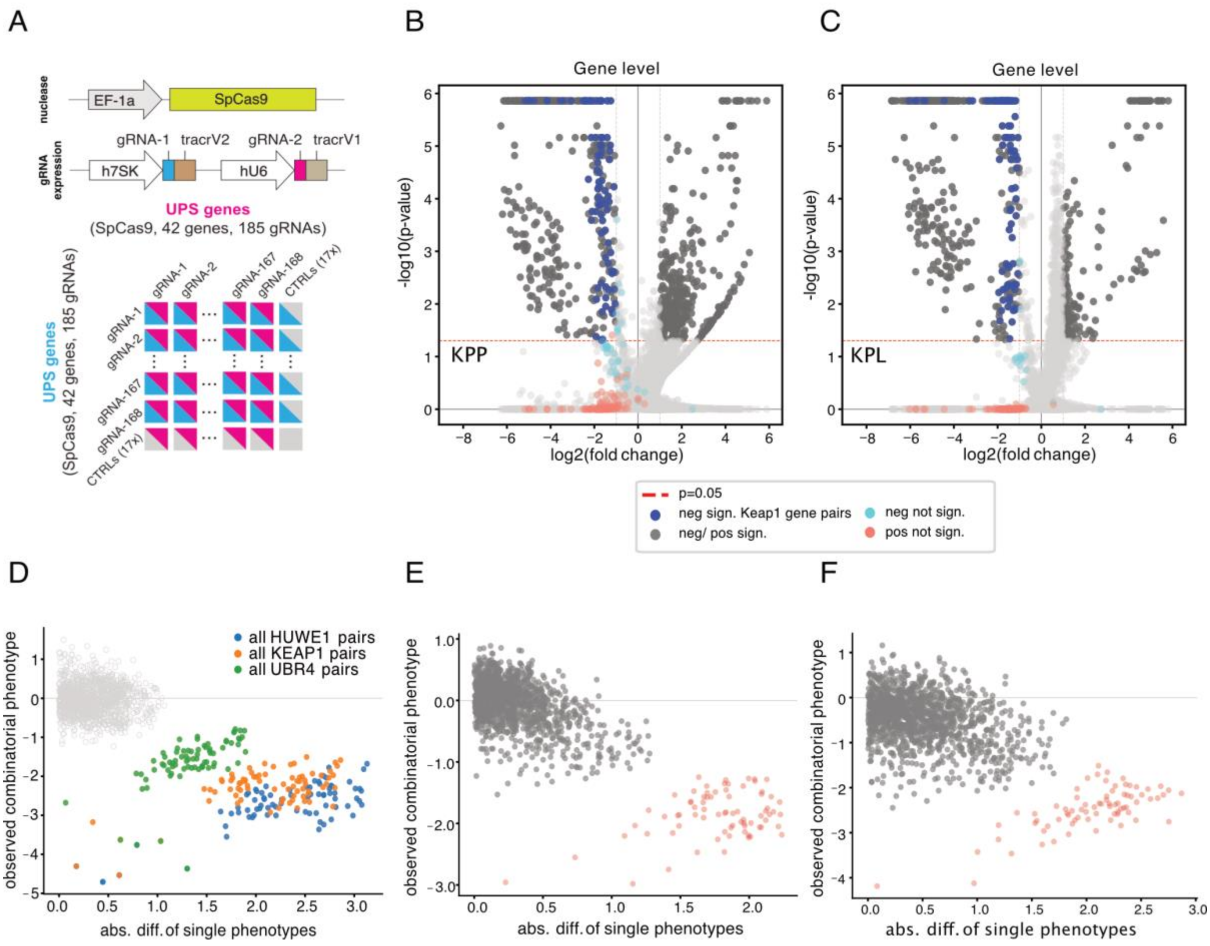

Appendix Figure S6: Multiplex CRISPR/Cas9 screens reveal keap1 dependent co-vulnerabilities.
A. UPS multiplex library design. Combinatorial gRNA constructs target two UPS genes simultaneously. The UPS multiplex library consists of two gRNA-expression cassettes; one side (h7SK) with 185 gRNAs targeting 46 genes (four gRNAs per gene), and the other side (hU6) with 185 gRNAs targeting 46 genes (four gRNAs per gene), resulting in 34255 gRNA combinations/ gene pairs.
B, C. Volcano plots of MAGeCK-derived LFCs and P-values for the multiplex dropout viability screen in KPP (B) and KPL (C). FDR for positive and negative selections are color-coded grey. Keap1 dependent gene pairs are highlighted in blue. Significant ($P < 0.05$) data points with LFC > 1 or LFC < -1 have dashed strokes.
D-F. Compare the difference between the single phenotypes and the combinatorial phenotypes in KP (D), KPP (E) and KPL (F) cell lines. The highlighted dots in pink color denote KEAP1 combinations in E and F.

Appendix Figure S7:

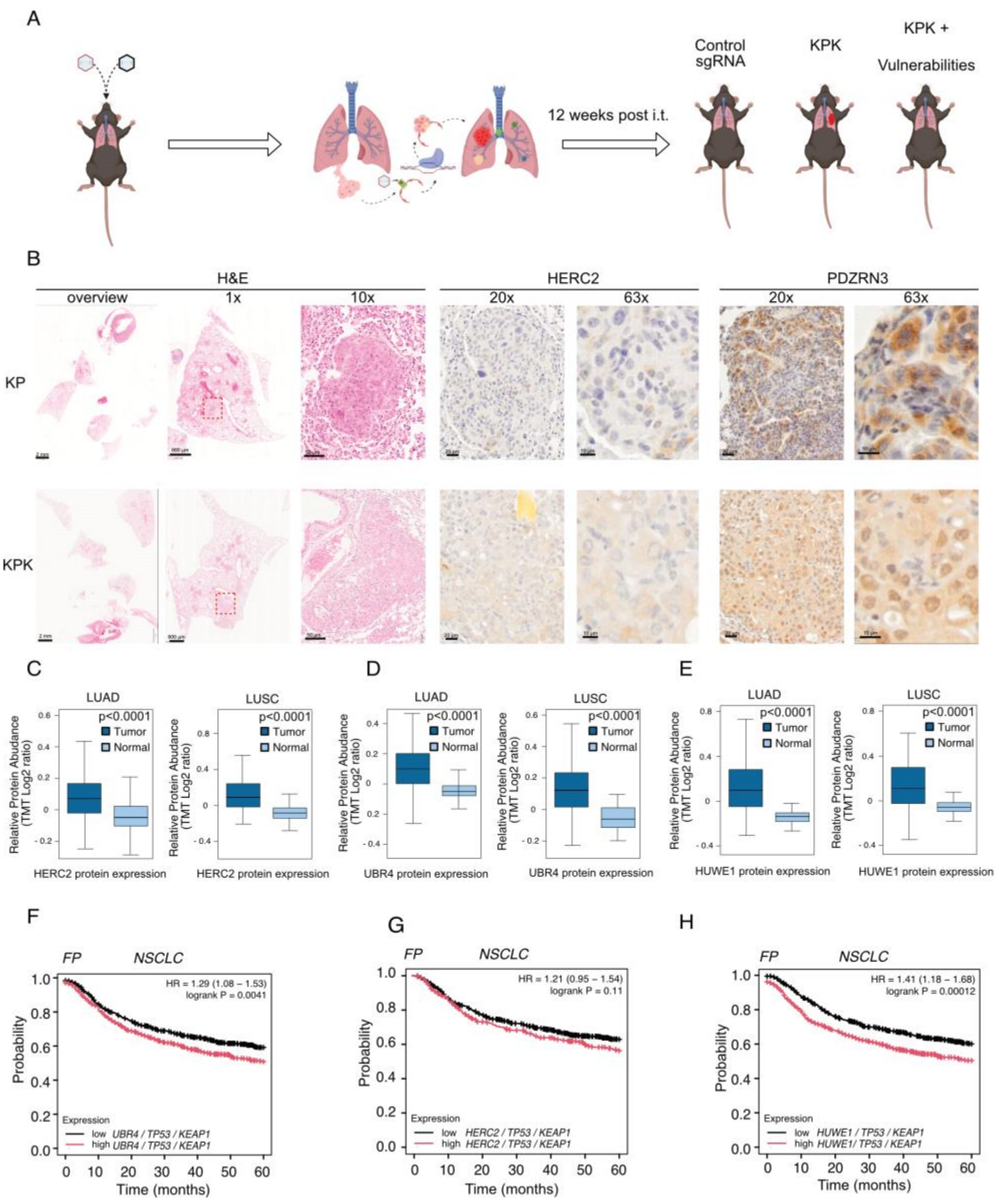

Appendix Figure S7: HERC2 and PDZRN3 displays increased expression in lung tumors upon oncogenic transformation.

A. Schematic representation of intratracheal tumor induction *via* CRISPR/Cas9.

B. Representative H&E sections of mice 12 weeks post intratracheal intubation with various genetic combinations; KP=KrasG12D:Tp53; KPK=KrasG12D:Tp53:Keap1. Representative IHC of HERC2 and PDZRN3 in KP and KPK tumors. Scale bar, 1/2 mm, 10/50/100 µm.

C - E. Publicly available expression data of Herc2 (B), UBR4 (C) and HUWE1(D) in NSCLC, relative to adjacent non-transformed tissue. Data obtained from https://cprosite.ccr.cancer.gov.

F- H. Kaplan-Meier curves of five-year progression free survival (FP) rates of lung cancer patients based on TP53, KEAP1, with UBR4 (F), HERC2 (G) and HUWE1 (H) gene expression levels. Kmplot.com.
