# Supplementary material for: Targeting ubiquitin signaling vulnerabilities in KEAP1-inactivated lung cancer
Source: EMBO J. 2026 Mar 20;45(9):3276–305. doi: 10.1038/s44318-026-00737-9 (PMC13144482; doi:10.1038/s44318-026-00737-9)
Supplement: Supplementary file 9 — Expanded View Figures [file 44318_2026_737_MOESM9_ESM.pdf]

Expanded View Figures

Figure EV1. Generation of UPS-centric single CRISPR/Cas9 library.

(**A**) Lorenz curve displaying the cumulative fraction of represented NGS reads versus the gRNAs ranked by abundance of each library revealed a uniform distribution of gRNA sequences. Area under the curve values (AUC) confirm the uniform gRNA distribution of these libraries. (**B**) The E3-DUB gRNA single screens are highly reproducible, as visualized by scatter plots comparing biological replicates (Exp#1/Exp#2/Exp#3) of normalized gRNA read counts at the 14-day time point in single CRISPR/Cas9 screens. (**C**, **D**) Sequencing depth of library and 14-day time point for gRNA (**C**) and gene level (**D**) plotted. Sample minimas are defined as the lowest single black dot, medians as green lines, bounds of boxes and whiskers as 25% of the sample population. Sample size (n) is three and indicated as the number associated with cell line IDs (e.g., KP1, KP2, and KP3). (**E**) Schematics of single and multiplex CRISPR/Cas9 screens. For a single library- coverage 1000x, MOI of 0.5 and for a multiplex library- coverage 60x and MOI of 0.5.

© The Author(s)

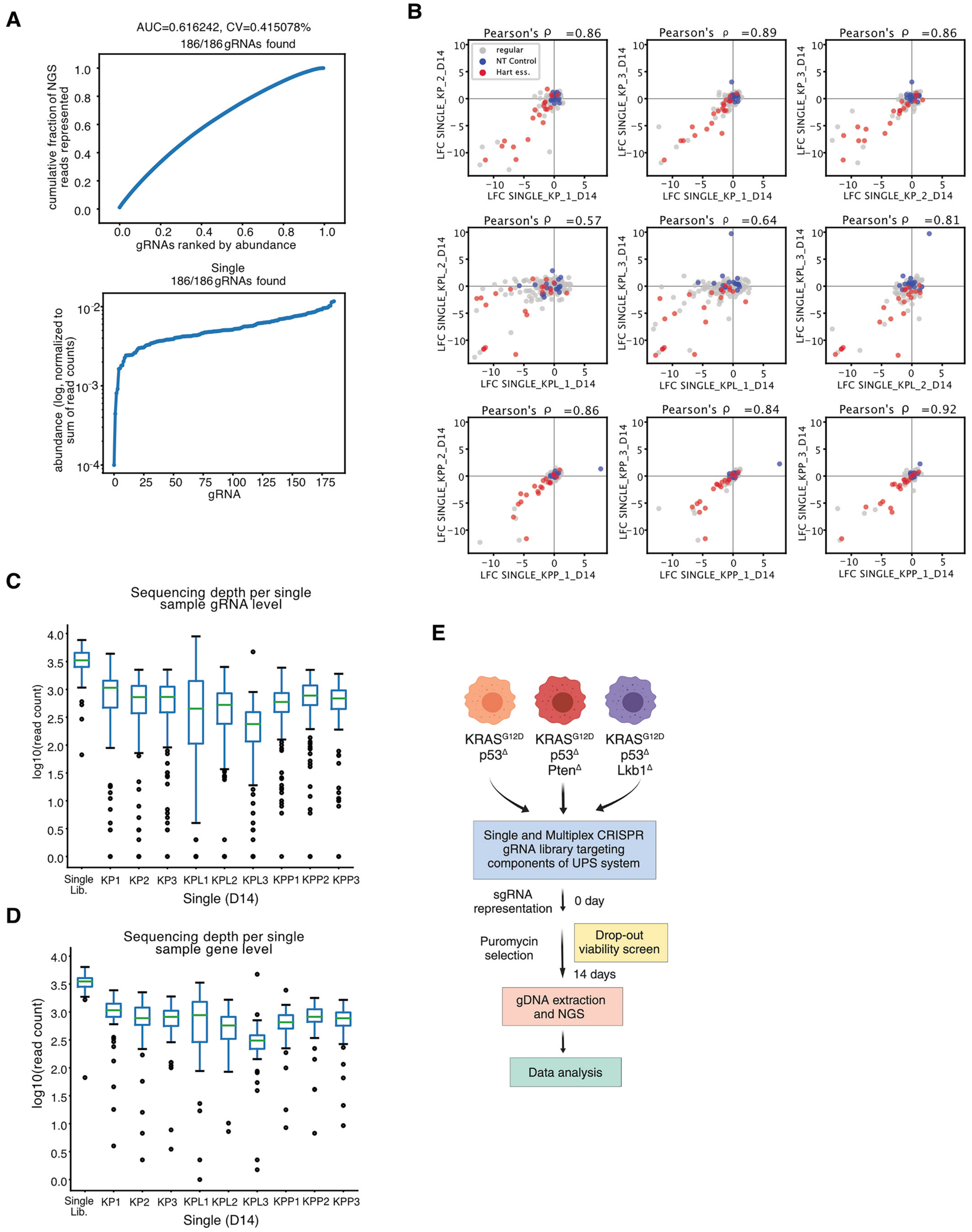
A
AUC=0.616242, CV=0.415078%
186/186 gRNAs found
cumulative fraction of NGS reads represented
gRNAs ranked by abundance
Single
186/186 gRNAs found
abundance (log, normalized to sum of read counts)
gRNA
B
Pearson's ρ =0.86
Pearson's ρ =0.89
Pearson's ρ =0.86
regular
NT Control
Hart ess.
LFC SINGLE_KP_2_D14
LFC SINGLE_KP_3_D14
LFC SINGLE_KP_1_D14
LFC SINGLE_KP_2_D14
Pearson's ρ =0.57
Pearson's ρ =0.64
Pearson's ρ =0.81
LFC SINGLE_KPL_2_D14
LFC SINGLE_KPL_3_D14
LFC SINGLE_KPL_1_D14
LFC SINGLE_KPL_2_D14
Pearson's ρ =0.86
Pearson's ρ =0.84
Pearson's ρ =0.92
LFC SINGLE_KPP_2_D14
LFC SINGLE_KPP_3_D14
LFC SINGLE_KPP_1_D14
C
Sequencing depth per single sample gRNA level
log10(read count)
Single Lib.
KP1
KP2
KP3
KPL1
KPL2
KPL3
KPP1
KPP2
KPP3
Single (D14)
D
Sequencing depth per single sample gene level
log10(read count)
Single (D14)
E
KRAS^{G12D} p53$^{\Delta}$
KRAS^{G12D} p53$^{\Delta}$ Pten$^{\Delta}$
KRAS^{G12D} p53$^{\Delta}$ Lkb1$^{\Delta}$
Single and Multiplex CRISPR gRNA library targeting components of UPS system
sgRNA representation
0 day
Puromycin selection
Drop-out viability screen
14 days
gDNA extraction and NGS
Data analysis

 © The Author(s)

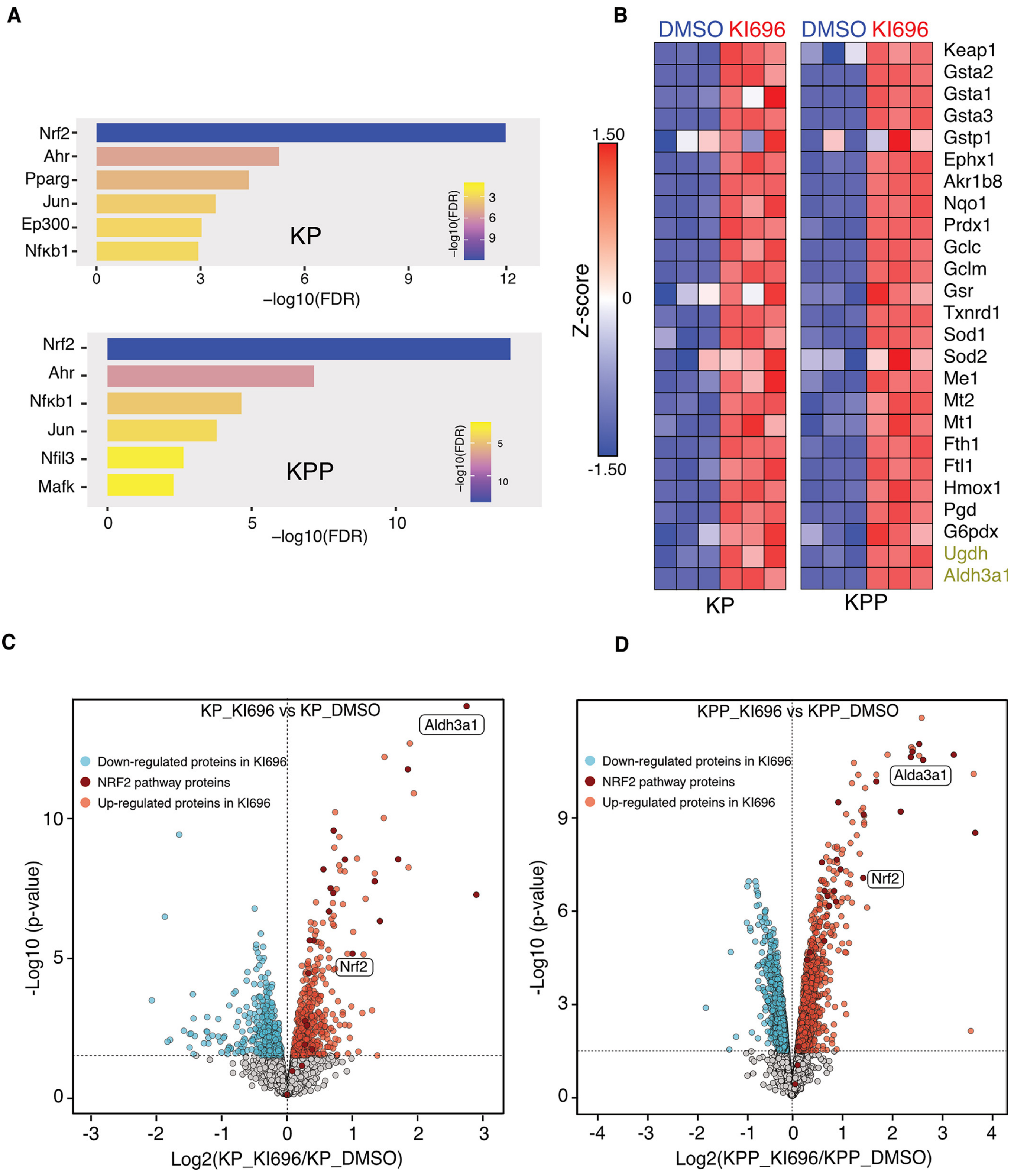
A
Nrf2
Ahr
Pparg
Jun
Ep300
Nfκb1
KP
−log10(FDR)
Nrf2
Ahr
Nfκb1
Jun
Nfil3
Mafk
KPP
−log10(FDR)
B
DMSO KI696 DMSO KI696
Z-score
1.50
0
-1.50
Keap1
Gsta2
Gsta1
Gsta3
Gstp1
Ephx1
Akr1b8
Nqo1
Prdx1
Gclc
Gclm
Gsr
Txnrd1
Sod1
Sod2
Me1
Mt2
Mt1
Fth1
Ftl1
Hmox1
Pgd
G6pdx
Ugdh
Aldh3a1
KP
KPP
C
KP_KI696 vs KP_DMSO
Aldh3a1
Down-regulated proteins in KI696
NRF2 pathway proteins
Up-regulated proteins in KI696
Nrf2
-Log10 (p-value)
Log2(KP_KI696/KP_DMSO)
D
KPP_KI696 vs KPP_DMSO
Alda3a1
Down-regulated proteins in KI696
NRF2 pathway proteins
Up-regulated proteins in KI696
Nrf2
-Log10 (p-value)
Log2(KPP_KI696/KPP_DMSO)

Figure EV2. Keap1 inhibition activates NRF2-driven pathways.

(**A**) KP (top) and KPP (bottom) cells were treated with KI696 (1 μM) for 24 h, and changes in gene expression were assessed by RNA-seq. Results are presented as enriched transcription factor signatures. (**B**) Heat map showing gene expression (*z*-score) of NRF2 target genes in *DMSO* and KI696 ($n = 3$) treated KP (left) and KPP (right) cells. (**C**, **D**) Whole-cell proteome analysis by mass spectrometry. Volcano plot of proteomic changes in KP cells (**C**) or KPP cells (**D**) treated with KI696 (1 μM) versus DMSO. Significantly decreased (blue) and increased (red) proteins ($p < 0.05$) are shown; dark red highlights significantly increased NRF2 pathway proteins ($n = 3$). Statistical significance was assessed by two-sided moderated t-test as implemented in the limma package via FragPipe Analyst; *p* values were adjusted using the Benjamini–Hochberg method.

 © The Author(s)

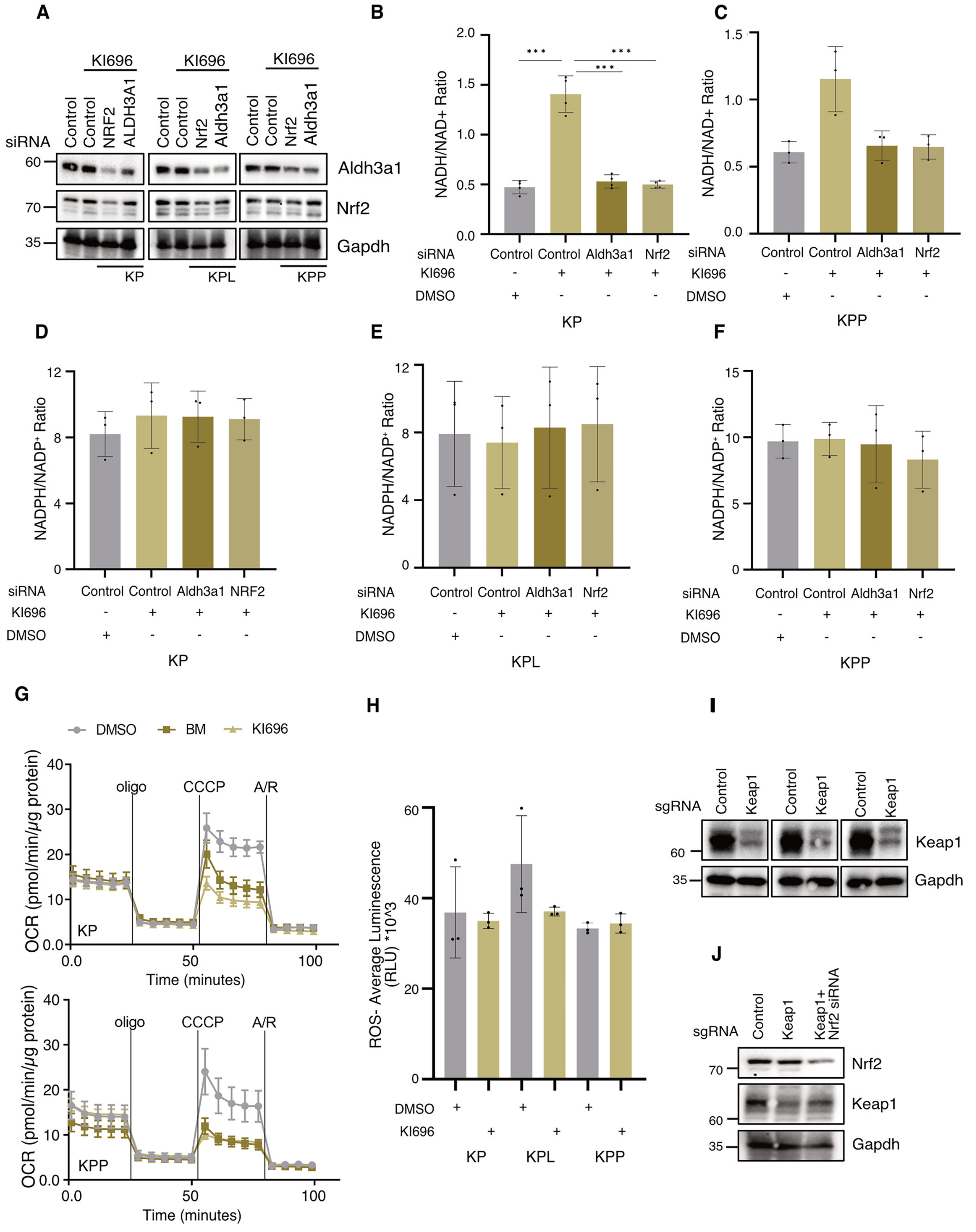
A
B
C
D
E
F
G
H
I
J
KI696
siRNA
Control
NRF2
ALDH3A1
Aldh3a1
Nrf2
Gapdh
KP
KPL
KPP
NADH/NAD+ Ratio
NADPH/NADP+ Ratio
KI696
DMSO
DMSO
BM
OCR (pmol/min/μg protein)
Time (minutes)
oligo
CCCP
A/R
ROS- Average Luminescence (RLU) *10^3
sgRNA
Keap1
Keap1+ Nrf2 siRNA

Figure EV3. Keap1 regulates redox stress and mitochondrial functions.

(**A**) Immunoblots for Aldh3a1, NRF2, and GAPDH in KP, KPL, and KPP cells transfected with si-Control, si-NRF2, or si-Aldh3a1, in the presence or absence of KI696. (**B**) NADH/NAD^+ ratio in KP cells treated with control, Aldh3a1, or NRF2 siRNA, in the presence of DMSO or KI696. Data represent mean ± SD of three biological replicates. ***$P < 0.001$. Control vs control + KI696 $P = 0.0001$, Control + KI696 vs Aldh3a1 si + KI696 $P = 0.0001$, Control + KI696 vs NRF2 si + KI696 $P = 0.0001$. Statistical significance was calculated using one-way ANOVA. (**C**) NADH/NAD^+ ratio in KPP cells treated with control, Aldh3a1, or NRF2 siRNA, in the presence of DMSO or KI696. Data represent mean ± SD of three biological replicates. ns not significant ($P > 0.05$). Statistical significance was calculated using one-way ANOVA. (**D–F**) NADPH/$NADP^+$ ratio in KP (**D**), KPL (**E**), or KPP (**F**) cells treated with control, Aldh3a1, or NRF2 siRNA, in the presence of DMSO or KI696. Data represent mean ± SD of three biological replicates. ns not significant ($P > 0.05$); statistical significance determined by one-way ANOVA. (**G**) Oxygen consumption rate (OCR) of KP (top) and KPP (below) cells treated with DMSO, KI696, or Bardoxolone methyl (BM). Data represent mean ± SEM of five technical replicates. (**H**) ROS levels of KP, KPL and KPP cells treated with DMSO or KI696 for 48 h. ns not significant ($P > 0.05$). Statistical significance was calculated using a paired *t*-test. (**I**) Immunoblots for Keap1 and GAPDH in KP, KPL, and KPP cells transduced with Control or Keap1 sgRNA. (**J**) Immunoblots for NRF2, Keap1, and GAPDH in KPP cells transduced with Control sgRNA, Keap1 sgRNA, or Keap1 sgRNA + NRF2 siRNA.

 © The Author(s)

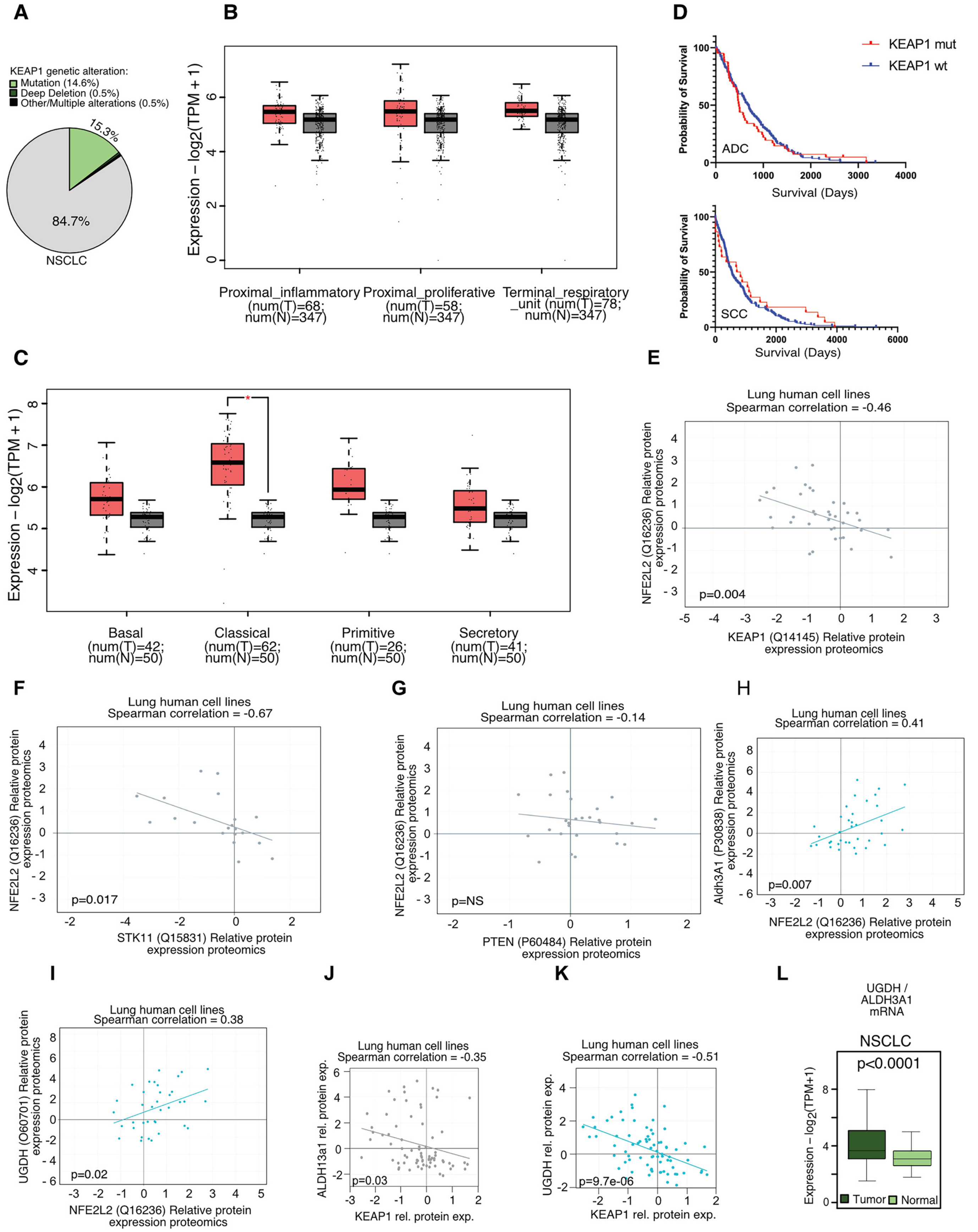
A
B
C
D
E
F
G
H
I
J
K
L
KEAP1 genetic alteration:
Mutation (14.6%)
Deep Deletion (0.5%)
Other/Multiple alterations (0.5%)
15.3%
84.7%
NSCLC
Expression – log2(TPM + 1)
Proximal_inflammatory (num(T)=68; num(N)=347)
Proximal_proliferative (num(T)=58; num(N)=347)
Terminal_respiratory _unit (num(T)=78; num(N)=347)
KEAP1 mut
KEAP1 wt
Probability of Survival
ADC
SCC
Survival (Days)
Basal (num(T)=42; num(N)=50)
Classical (num(T)=62; num(N)=50)
Primitive (num(T)=26; num(N)=50)
Secretory (num(T)=41; num(N)=50)
Lung human cell lines
Spearman correlation = -0.46
NFE2L2 (Q16236) Relative protein expression proteomics
KEAP1 (Q14145) Relative protein expression proteomics
p=0.004
Lung human cell lines
Spearman correlation = -0.67
STK11 (Q15831) Relative protein expression proteomics
p=0.017
Lung human cell lines
Spearman correlation = -0.14
PTEN (P60484) Relative protein expression proteomics
p=NS
Lung human cell lines
Spearman correlation = 0.41
Aldh3A1 (P30838) Relative protein expression proteomics
NFE2L2 (Q16236) Relative protein expression proteomics
p=0.007
Lung human cell lines
Spearman correlation = 0.38
UGDH (O60701) Relative protein expression proteomics
NFE2L2 (Q16236) Relative protein expression proteomics
p=0.02
Lung human cell lines
Spearman correlation = -0.35
ALDH13a1 rel. protein exp.
KEAP1 rel. protein exp.
p=0.03
Lung human cell lines
Spearman correlation = -0.51
UGDH rel. protein exp.
KEAP1 rel. protein exp.
p=9.7e-06
UGDH / ALDH3A1 mRNA
NSCLC
p<0.0001
Expression – log2(TPM+1)
Tumor
Normal

© The Author(s)

Figure EV4. Comparison of relative expression data.

(**A**) Occurrence of mutations within Keap1 in NSCLC adenocarcinoma and squamous cell carcinoma. Data from cbiportal.org. (**B**, **C**) Expression of KEAP1 in non-transformed (N) and (**B**) LUAD (T) or (**C**) LUSC (T) samples, represented according to consensus LUAD and LUSC subtypes. Publicly available data were extracted from TCGA via the web tool GEPIA2 (http://gepia2.cancer-pku.cn/). Normal samples comprises TCGA normal and GETEx samples. Num indicates samples analysed. Significance was calculated with Dixon's *Q*-test. *P* value $^*p < 0.05$. The box plot represents the lower (25%) and upper quartile (75%) with the median (50%) displayed. The whisker represents the minimum and maximum. (**D**) Patient survival data based on Keap1 status (wildtype or mutant) in ADC (top) (NS) and SCC (bottom) NS not significant ($P > 0.05$). (Source: Tcga, Xena variant. https://xena.ucsc.edu/). (**E**) Correlation of protein expression of NFE2L2 and Keap1 in lung cancer cell lines. (**F**) Correlation of protein expression of STK11 and Keap1 in NSCLC cancer cell lines. (**G**) Correlation of protein expression of NFE2L2 and PTEN in lung tumors. (**H**, **I**) Correlation of protein expression of NFE2L2 and Aldh3a1 (**H**) and UGDH (**I**) in lung tumor cancer cell lines. (**J**, **K**) Keap1 protein expression relative to Aldh3A1 (**J**) and UGDH (**K**), respectively. (**L**) UGDH and ALDH3A1 gene expression levels in lung cancer samples relative to adjacent non-transformed tissue. *P* value $^*p < 0.0001$. The significance was calculated with a one-way ANOVA comparing tumors with normal samples. Data from gepia2.cancer-pku.cn.

 © The Author(s)

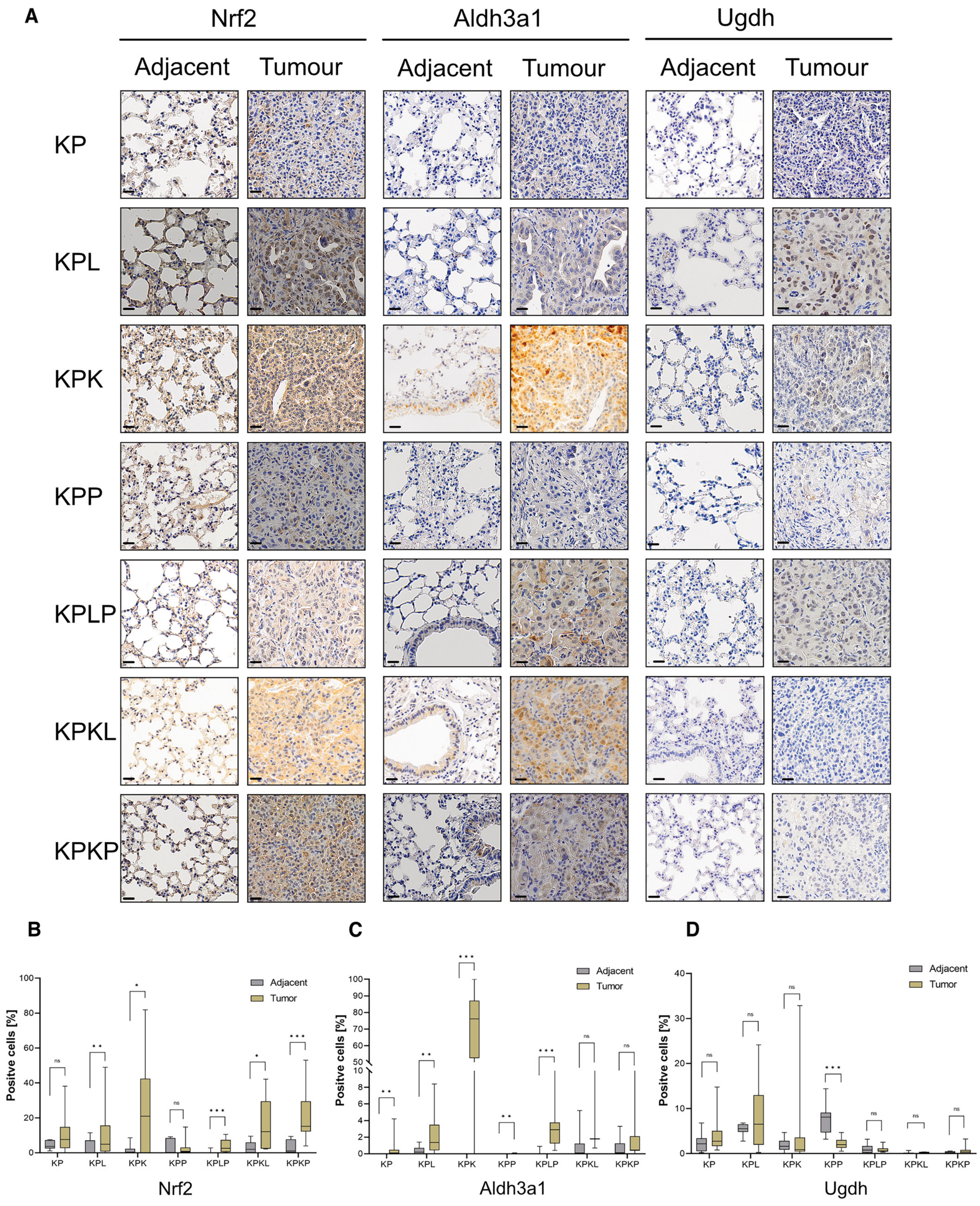
A
Nrf2
Aldh3a1
Ugdh
Adjacent
Tumour
KP
KPL
KPK
KPP
KPLP
KPKL
KPKP
B
C
D
Adjacent
Tumor
Positive cells [%]
KP
KPL
KPK
KPP
KPLP
KPKL
KPKP
Nrf2
Aldh3a1
Ugdh

Figure EV5. Immunohistochemistry (IHC) analysis of reductive stress markers in tumors and adjacent lung tissue.

(**A**) Representative IHC sections from mice of different genetic backgrounds, 12 weeks post-intubation. Staining for NRF2, ALDH3A1, and UGDH in adjacent and tumor tissue was performed to assess redox stress. Scale bar, 20 µm. (**B–D**) Quantification of positive cells was performed using QuPath (v0.6.0) from IHC staining shown in (**A**) for Nrf2 (**B**), Aldh3a1 (**C**), and Ugdh (**D**). Significance was calculated using the Mann−Whitney test. (Significance $p < 0.05$) (n[adjacent] >8; n[tumor] >8; except n[KPKL) = 3). (**B**) p(KP) = 0.1216; p(KPL) = 0.0019; p(KPK) = 0.0430; p(KPP) = 0.2002; p(KPLP) = 0.0002; p(KPKL) = 0.0348; p(KPKP) < 0.0001. (**C**) p(KP) = 0.0013; p(KPL) = 0.0081; p(KPK) < 0.0001.; p(KPP) = 0.0044; p(KPLP) < 0.0001; p(KPKL) = 0.0824; p(KPKP) = 0.1670. (**D**) p(KP) = 0.2257; p(KPL) = 0.7694; p(KPK) = 0.3115; p(KPP) < 0.0001.; p(KPLP) = 0.8071; p(KPKL) = 0.0070; p(KPKP) = 0.4555. The box plot represents the lower (25%) and upper quartile (75%) with the median (50%) displayed. The whisker represents the minimum and maximum.

© The Author(s)
